# Cystoscopic depth estimation using gated adversarial domain adaptation

**DOI:** 10.1007/s13534-023-00261-3

**Published:** 2023-01-20

**Authors:** Peter Somers, Simon Holdenried-Krafft, Johannes Zahn, Johannes Schüle, Carina Veil, Niklas Harland, Simon Walz, Arnulf Stenzl, Oliver Sawodny, Cristina Tarín, Hendrik P. A. Lensch

**Affiliations:** 1 Institute for System Dynamics, University of Stuttgart, Stuttgart, Germany; 2 Institute for Computer Graphics, University of Tübingen, Tübingen, Germany; 3 Urology Clinic, University Hospital of Tübingen, Tübingen, Germany

**Keywords:** Neural networks, Domain adaptation, Depth estimation, Endoscopy, Synthetic data

## Abstract

Monocular depth estimation from camera images is very important for surrounding scene evaluation in many technical fields from automotive to medicine. However, traditional triangulation methods using stereo cameras or multiple views with the assumption of a rigid environment are not applicable for endoscopic domains. Particularly in cystoscopies it is not possible to produce ground truth depth information to directly train machine learning algorithms for using a monocular image directly for depth prediction. This work considers first creating a synthetic cystoscopic environment for initial encoding of depth information from synthetically rendered images. Next, the task of predicting pixel-wise depth values for real images is constrained to a domain adaption between the synthetic and real image domains. This adaptation is done through added gated residual blocks in order to simplify the network task and maintain training stability during adversarial training. Training is done on an internally collected cystoscopy dataset from human patients. The results after training demonstrate the ability to predict reasonable depth estimations from actual cystoscopic videos and added stability from using gated residual blocks is shown to prevent mode collapse during adversarial training.

## Introduction

Depth, or distance, information from a sensor is paramount for localization and mapping algorithms, especially when using cameras as the main sensor modality. For this reason, current robotics applications combine LIDAR, or similar, sensors to create what is known as an RGB-D (color and depth) camera image. This provides a dense (or at least partially dense) pixel-wise depth map for each given matching image. By using the camera’s intrinsic parameters, a point cloud of the scene can be re-projected and, through additional algorithms, the extrinsic position of the camera can be reconstructed while simultaneously mapping the environment. It is also possible to do this without the direct depth information by finding matching points in sequential images and using triangulation methods to accomplish the same task with more sparse data points.

When operating inside the human body, however, these methods are not as feasible and particularly during cystoscopic operations where the camera and instruments must fit through the urethra to reach the bladder, inclusion of depth measuring sensors like LIDAR are out of the question. These restrictions also make it more difficult for the physician to ensure that the entire bladder has been seen giving rise for the need of methods to map the bladder to ensure full visual coverage. While using stereo cameras provides a more robust triangulation-based depth reconstruction, for the same reason of restricted space this method is not feasible and using a monocular camera for depth estimation is unavoidable to obtain the same localization and mapping goals. An additional problem in the cystoscopic environment is that the scene may change fast enough that also using sequential image frames for pseudo-triangulation is not feasible due to difficulties in matching features between images. Some works have proposed ideas to circumvent these limitations, for example [1], but they require additional information, such as an underlying model, that is not always obtainable. For these reasons, monocular depth estimation remains a hot topic of research and when using a single image, one method has come to stand out: image domain adaptation.

This work leverages the idea that instead of measuring, the entire domain can be simulated, in this case a synthetic cystoscopic environment, including the desired output information of depth from the camera. This can be used to compensate for the missing information in a second domain: the real environment. Adaptation between different domains is generally possible when they are similar enough that there exists a feasible transfer function from one to the other. Generative Adversarial Networks (GANs) have shown this to be true by learning this transfer function using neural networks. Under the assumption that the synthetic domain can be constructed accurately enough to form a finite information gap from the real domain, this work aims to find the associated transfer function.

With this in mind, the training method proposed in [2] is used as a foundation and modified. The approach uses adversarial training to retrain an encoder from a encoder-decoder network such that it produces similar latent features from real images as from the synthetic images it was originally trained on. The encoder in this work is modified so that the domain adaptation occurs only in added residual blocks, not through retraining the entire encoder. This approach of using residual blocks for the additional learning was also taken in [3] for transfer learning to improve over comparative GAN approaches, but in this work it is directly applied to a GAN approach. In addition, learnable gates are included in the added layers to bring additional stability during the adversarial training by smoothly fading in domain specific features.

### Related work

Depth estimation from images is not a new field of study. Techniques such as *structure from motion* have been around since at least the 1970 s [4] and, more recently, augmented reality requires real-time estimation of this information. Until recently, the approaches using only camera images all relied upon using corresponding points between consecutive images. One state-of-the-art open source tool COLMAP [5] excels at this. Machine learning has recently been used to enhance results of these methods for more complete and continuous depth maps [6]. The downside to all of these existing techniques, however, is that they are not universal.

Domains that change quickly or lack distinct features between frames, such as endoscopic videos, render existing methods unusable. Therefore, newer techniques focus on methods to make this prediction without the need for distinct feature recognition. These techniques begin with a supervised approach, in which the problem can be seen as a regression problem given a color image as input and a ground truth depth map as output. Two frequently used neural network architectures that do this are DispNet [7] and fully convolutional residual networks (FCRN) [8]. One non-negligible difficulty with formulating the problem this way is the reliance on ground truth data. Unfortunately, the situations where only a camera is desired for extracting dense depth information are ones in which also using reliable distance sensors for ground truth is not feasible.

This dilemma led to more generalizable approaches capable of using synthesized data for supervised training and using domain adaptation to apply the results to real images. As already mentioned, AdaDepth [9] was the first to effectively do this and demonstrates the capability on various datasets of different domains. Shortly thereafter came works in the areas of colonoscopy [10] and bronchoscopy [2], where depth predictions in endoscopic surgeries could now be done. Both of these works used simplified organ reconstructions and phantom scans to create synthetic data with ground truth depth for initial neural network training before performing a domain adaptation to the real images. While these environments also suffer from the aforementioned deformability and working space problems, particularly the lungs and airways have the advantage that the general shape and images between different patients does not vary to the same extent as for the bladder. The bladder is one of the most deformable organs in the body and during surgeries, the fill level is continuously changed to allow for different views or cutting actions making the scene very dynamic. However, this does not mean that the approaches cannot be applied to the bladder, it has just not yet (to the authors’ knowledge) been done.

The contributions of this work can be summarized as

- the creation of a synthetic cystoscopic environment for rendering images and corresponding depth maps,
- the use of a modified encoder structure for more stabilized GAN training during domain adaptation,
- and evaluation of the prior two contributions on real, clinical endoscopic video data.

## Materials and methods

The training takes place in two parts. First, a neural network is trained on synthetic data to learn the mapping from synthetic images to depth maps and in the second step, gated residual blocks for domain transfer are inserted into the encoder and adversarial training is performed to adapt the encoder for real cystoscopic images. This section will outline the developed network structure, the data generation, and training methods used to accomplish this. First, the structure for depth estimation from synthetic images using an encoder-decoder network is explained. Following the synthetic training, the structure is modified for domain transfer from real to synthetic latent features through a modification of the encoder where gated residual blocks are inserted.

### Synthetic domain network structure

**Fig. 1:**
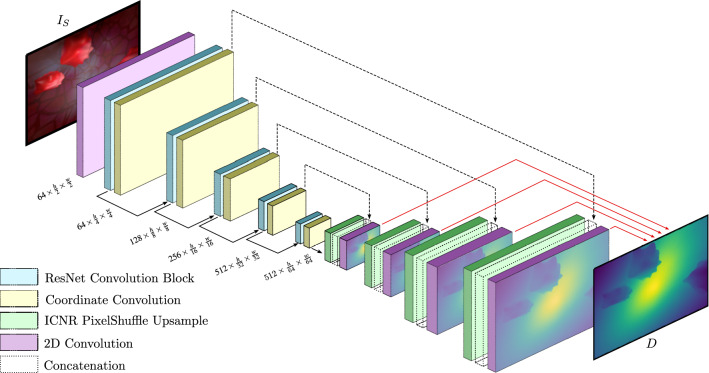
Synthetic data depth prediction network. Solid red arrows indicate the points at which the loss is calculated and include upsampling as needed to match the pixel dimensions of the ground truth depth image *D* (shown here in color for illustration purposes only)

**Fig. 2:**
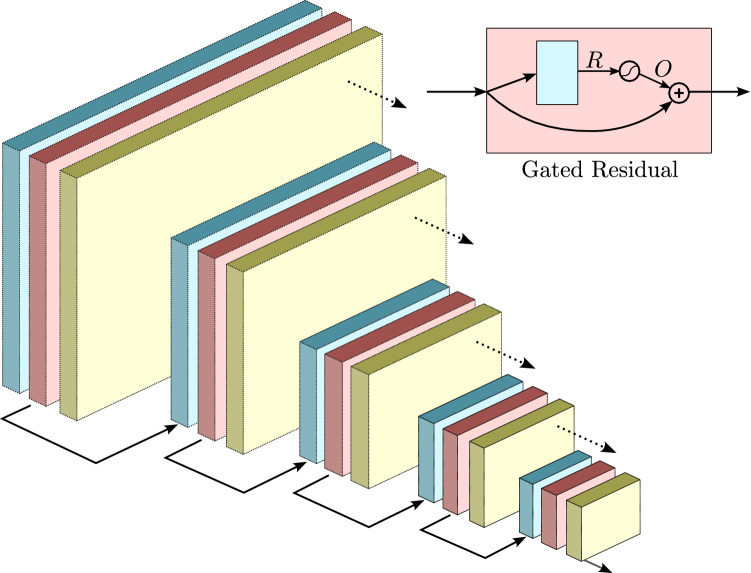
Modified encoder with gated residual blocks

The overall depth prediction network structure follows the U-Net in [2] with differences mainly in the decoder and the activation functions. Instead of simple nearest neighbor upsampling, the ICNR initialized sub-pixel convolution approach [11] is used. On real images this step drastically reduced checkerboard artifacts (from empirical testing). The resulting network (Figure 2) is the backbone for learning depth estimation from synthetically generated images. As seen in Fig. 1, the decoder is guided to predict depth at multiple levels during training. This encourages the latent features to include information about the depth. Once this network is trained with a standard supervised regression approach, the modifications outlined in the next section are made to handle the domain transfer learning for real cystoscopy images.

### Domain transfer network structure

While direct application of the synthetic depth prediction network on real images without any network modifications or re-training produces plausible depth maps, these are still subject to inaccuracy due to the domain shift (see third column in Fig. 10). This domain shift is handled through a domain transfer learned through generative adversarial training between a new encoder and multiple discriminators. However, rather than retrain the entire encoder, as is done in [2], which can lead to more unstable GAN training, a gated transfer learning approach is implemented with added residual blocks at each encoder level. These blocks are initially disabled as GAN training is started and the gates are slowly opened with a learned coefficient $\alpha _\textsf {i}$ for each encoder level $\textsf {i}$ using

1 $$\begin{aligned} O_\textsf {i}= R_\textsf {i}\circ \tan {\alpha _\textsf {i}}, \end{aligned}$$

where *R* and *O* are the outputs of the added ResNet block and the resulting gated output, respectively. This follows the same method as in [12] using the idea of ReZero from [13]. The intent here is that the residual blocks will learn how to correct for the domain shift and the rest of the already trained encoder is left frozen to maintain the image features that contain the depth information. The modified encoder with residual blocks is shown in Fig. 2.

### Synthetic domain data and training

Here, the methods for the first step of training depth estimation within the synthetic cystoscopy domain are outlined.

#### Data generation

The tool of choice for rendering images of the synthetic environment is extremely important and directly influences the quality of the results. The generated images should be as *real* as possible. The tool used in this work to create the synthetically rendered images is the 3D rendering software Blender [14], which also includes a python interface for automated generation of different scenes and camera positions.

Realism in synthetic images can be divided into three categories: photo, physical, and functional realism [15]. The first refers to whether a rendered image produces the same visual response as a real scene. Physical realism is achieved when a synthetic image produces the same visual stimulation as a real scene. This is harder to achieve than photo realism and requires the render engine to accurately and realistically calculate the spectral properties of the light, observed at the viewpoint. Functional realism requires an image to contain the same visual information as a real scene. Hence, the observer must be able to extract the relevant properties such as sizes, shapes, motions, positions, and materials. This does not require the image to be physically realistic. For example, technical drawings can provide functional realism. For the task of object detection, it was found that a high level of photo realism is not required for high performance [16]. While this was shown for the task of object detection it is unknown for other tasks, such as monocular depth estimation.

**Fig. 3:**
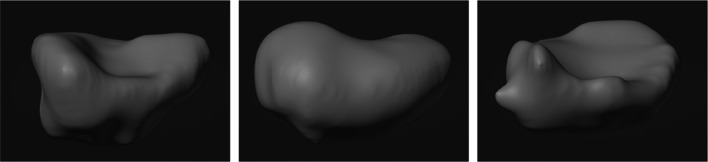
Anatomically accurate 3D bladder models with different filling states obtained by CT scans

The scene lighting has a drastic effect on physical cues for depth estimation in an endoscopic environment, which was a driving factor in [10]. The light source within an endoscopic environment is typically attached to the camera and, therefore, moves along with it. To capture illumination effects as best as possible, ray trace rendering is preferred over rasterization for creating synthetic images in order to model the light transport accurately capturing effects such as shadowing in a realistic way. Additionally, for depth estimation, the general shapes and sizes of objects need to be accurately represented. For this, all models need to be created within the bounds of physically possible features seen during a cystoscopy. This is accomplished by utilizing reconstructions of actual patient bladders taken from CT scans, a 3D imaging technique, from the study [17]. Examples are seen in Fig. 3 where it is also possible to see that the human bladder is a very irregularly shaped organ as the only consistent feature between the scans is that they are singular, closed volumes. The lights are simulated as two conical light sources placed on each side of the camera, similar to [10], to simulate a typical endoscope.

**Fig. 4:**
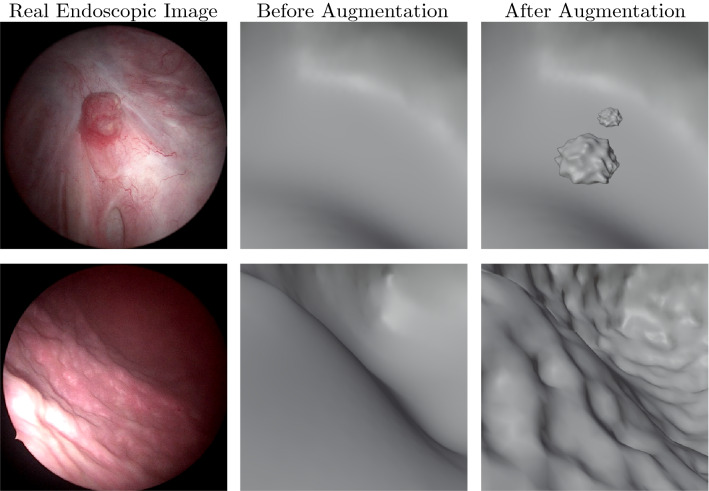
Bladder model geometry augmentations. The 3D bladder models are modified to cover tissue effects such as: (top) polyps, (bottom) bumpy bladder walls. Left image shows general tissue effect to be simulated, middle image shows model before augmentation, right image shows model after augmentation with either added bodies or added Perlin noise displacement. Note: the model images here are rendered using Phong shading, so it only appears that the simulated polyp is floating in space even though it is not

Due to the voxel resolution of the scanning method that generated the models the resulting models’ surfaces needed to be smoothed. Features such as divercula or polyps are, therefore, not represented. In addition, the walls in an actual bladder tend to be more wrinkly. To account for these missing features, additional geometry modifications are performed to randomly add fake polyps and a Perlin noise displacement texture across the model’s surface. Examples of these modifications are shown in Fig. 4 next to similar real images.

**Fig. 5:**
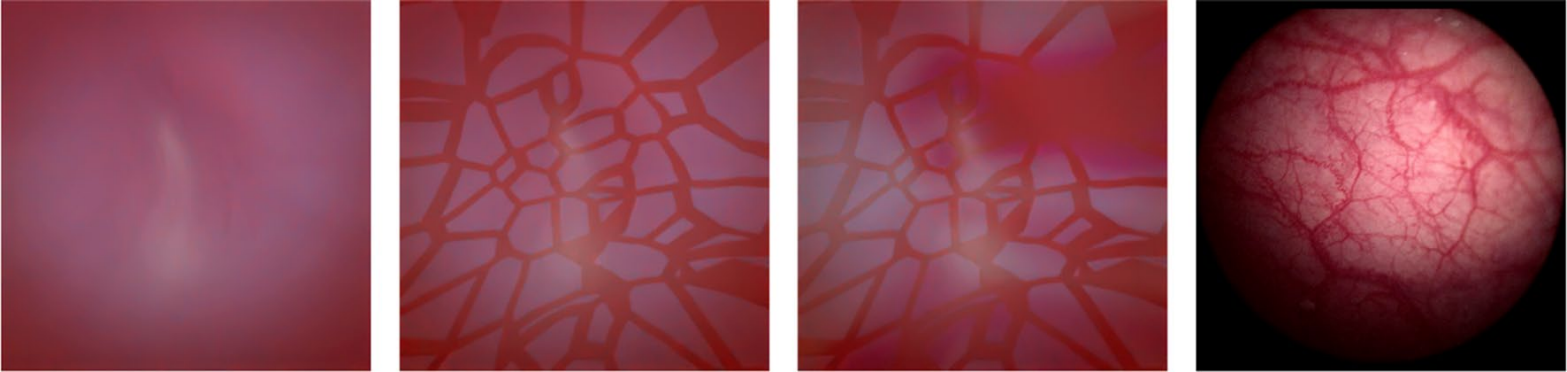
Bladder texture modifications left to right: Bladder base color, artificial blood vessels, artificial vessels and randomized texture brightness values, and real image of blood vessels for comparison

To avoid poor generalization due to the uniform texturing (default blender material) shown in Fig. 4, additional materials are used to represent a closer color representation to the real images including blood vessel-like structures. Translucent subsurface scattering is also enabled for this texture to better represent the optical properties of human tissue. A final touch of randomly generated texture brightness helps to make the model learn the difference between the reflective properties and shadows. These modifications are shown in Fig. 5.

**Fig. 6:**
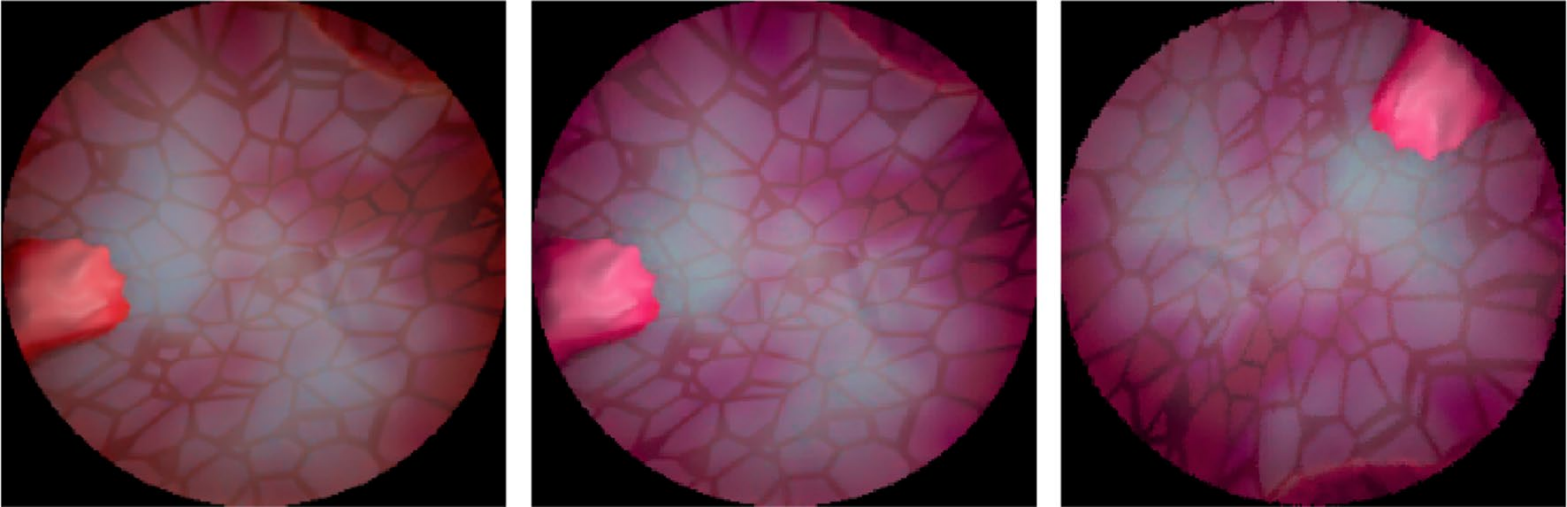
Data augmentation examples left to right: No augmentation, color jitter, and color jitter with rotation

Camera pose generation for the rendered images is a simple procedure since the bladder is a closed sphere-like volume. Vectors from the center of volume are randomly generated and a randomized distance from the intersection of the bladder wall provides the position of the camera. The viewing direction is then varied up to 30$\circ$ from the intersecting vector. The final augmentations come post rendering in the form of more standard image modification methods. These include: black circular mask generation, random color jitters, random translations, and random rotations from 0$\circ$ to  360$\circ$. The circular black mask is necessary as this information cannot be removed in the real images. Samples of these are seen in Fig. 6. Simultaneously to the color image rendering, depth maps are rendered out and matching transformation augmentations are applied accordingly.

#### Supervised training

The training for the synthetic domain is very straightforward. The goal of the network is to predict a depth map $D^*$ for a given synthetic image $I_S$. It is a standard supervised learning problem with the caveat that the depth loss is calculated at each level of the multi-resolution decoder. In order to do this at the pixel level, the same technique as in [2] is used, namely upsampling with bilinear interpolation, to reach the ground truth depth image *D* dimensions. The BerHu loss [18]

2 $$\begin{aligned} L_{\text {BerHu}}(D, D^*) = {\left\{ \begin{array}{ll} \left| D-D^* \right| &{} \text {if} \; \left| D-D^* \right| \le c \\ \frac{ \left( D-D^* \right) ^2+c^2}{2c} &{}\text {if} \; \left|D-D^*\right| > c \end{array}\right. } \end{aligned}$$

is used as it has been shown to outperform standard regression losses such as the $L_1$ or $L_2$ loss. The threshold $c=\frac{1}{5} \max _i \left( \left|D_i-D_i^*\right| \right)$ is defined as a fixed fraction of the maximum absolute difference for any pixel between the ground truth and prediction. Since the depth maps should be locally similar as the tissue is generally smooth and connected (excluding situations such as occlusion), an additional loss is calculated, namely a gradient loss

3 $$\begin{aligned} L_{\text {grad}} = \frac{1}{|N|} \sum _{i\in N} \left( \left( \nabla _x y_i \right) ^2 + \left( \nabla _y y_i \right) ^2 \right) , \end{aligned}$$

where $y_i= \log D_i - \log D^{*}_i$, and $\nabla _x$ and $\nabla _y$ denote the image gradients in horizontal and vertical directions for the number of valid pixels *N*. The loss term penalizes high image gradients of the difference between the prediction and ground truth in log scale. This produces more accurate gradients in the depth prediction without degrading the *L*2 regression loss [19].

The total resulting loss for the synthetic domain training is given as

4 $$\begin{aligned} L(D, D^*) = \sum _{l=1}^4 c_0 \, L_{\text {BerHu}}(D,u(D^*_l)) + c_1 \, L_{\text {grad}}(D,u(D^*_l)) \end{aligned}$$

with sensitivity tuning coefficients $c_0$ and $c_1$ between the two loss components. The individual loss components are summed across each decoder level with $l=4$ as the lowest resolution decoder output. Here, *u* is the bilinear interpolation upsampling to reach the image resolution of *D*.

### Domain transfer data and training

As is done for the synthetic domain, first an overview of the data used is provided, followed by the training procedure for accomplishing the task.

#### Data acquisition

The dataset for domain adaptation consists of 17 standard cystoscopic videos with an average frame rate of 25 frames per second. The videos consist both of normal diagnostic checks and trans-urethral resections of tumors. Most of these videos are recorded using analog equipment so before processing, a standard deinterlacing algorithm YADIF is run on the associated videos. The videos are then sampled every 5 frames to generate the initial raw data set.

**Fig. 7:**
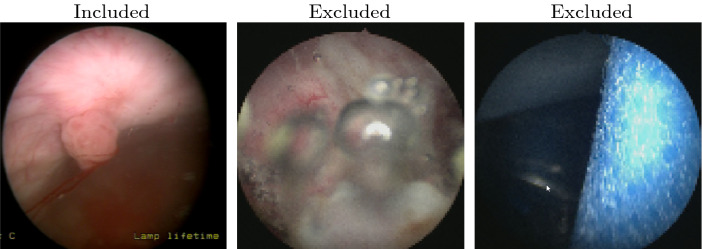
Frame selection from the clinical cystoscopy dataset. From left to right: included image for training, excluded blurry image, and excluded camera outside the body

Before the images can be used they are filtered to exclude irrelevant ones including: when the endoscope is outside the body, over exposure, and the image is too blurry or dark. This process is automated by first finding a fitting circular mask and then using tools such as a red threshold (for inside the bladder), Laplacian variance (blurriness), and a general brightness threshold. Further, more advanced filtering could be done including using a neural network classifier to exclude images with bubbles as these are not a part of the actual depth of the scene. Figure 7 shows some examples of excluded and included cystoscopic images.

#### Adversarial training

**Fig. 8:**
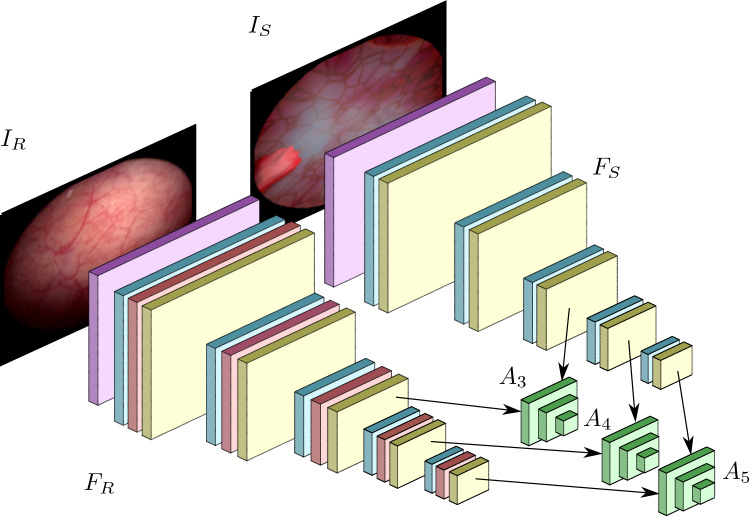
Adversarial domain adaptation scheme similar to that in [2]. The encoder $F_R$ for the real domain is initialized with weights from $F_S$ and includes the added residual blocks. Adversarial training is then performed where $F_R$ acts as a conditional generator that takes image $I_R$ as input. Discriminators $A_*$ are applied at the skip connections and trained to distinguish $F_S(I_S)$ from $F_R(I_R)$

After the network is trained to predict depth from the synthetic images, the network weights are frozen. Two copies of the encoder will be used during adversarial training: one $F_S$ is left unchanged and the other $F_R$ receives the gated residual blocks for domain transfer. It is worth noting here that the batch normalization statistics throughout the encoder are also frozen. This decision comes from a separate experimental investigation that found by doing this a better overall depth error was achieved after adversarial training. The training approach here follows the scheme in [2] where the decoder is not included in the adversarial training and instead the encoder is forced to learn similar latent vectors at the lower three levels as those output from the synthetic training. This is shown in Fig. 8. The individual discriminators $A_i$ with $i \in {3,4,5}$ also use the same PatchGAN structure as in [2].

The standard GAN training as proposed in [20] is used with the adversarial objective function

5 $$\begin{aligned}{} & {} L_A= \sum _{i\in {3,4,5}} \mathbb {E}_{I_S\sim X_S}[\log A_i \left( F_{Si} \left( I_S \right) \right) ] \nonumber \\{} & {} \quad\quad + \mathbb {E}_{I_R \sim X_R} [{\log (1- (A_{i}({F_{Ri}}({I_{R}}))))}] \end{aligned}$$

where $I_R$ is an image from the real domain and $I_S$ an image from the synthetic.

## Results

For the synthetic data, 5000 images per bladder model and material (textured and non-textured) were rendered. Therefore, 10000 images per bladder model are available with randomized viewpoints, viewing direction, light intensity, etc. Two of the 38 bladder models are used each for the validation and test sets. This amounts to 340000 images for the train set ($90\%$) and 20000 images for both the test and validation set ($5\%$ each). For the domain adaptation, approximately 16600 real cystoscopy images without ground truth labels are used. The images from both datasets are fed to the network at their original resolution of $256\times 256$ pixels.

**Fig. 9:**
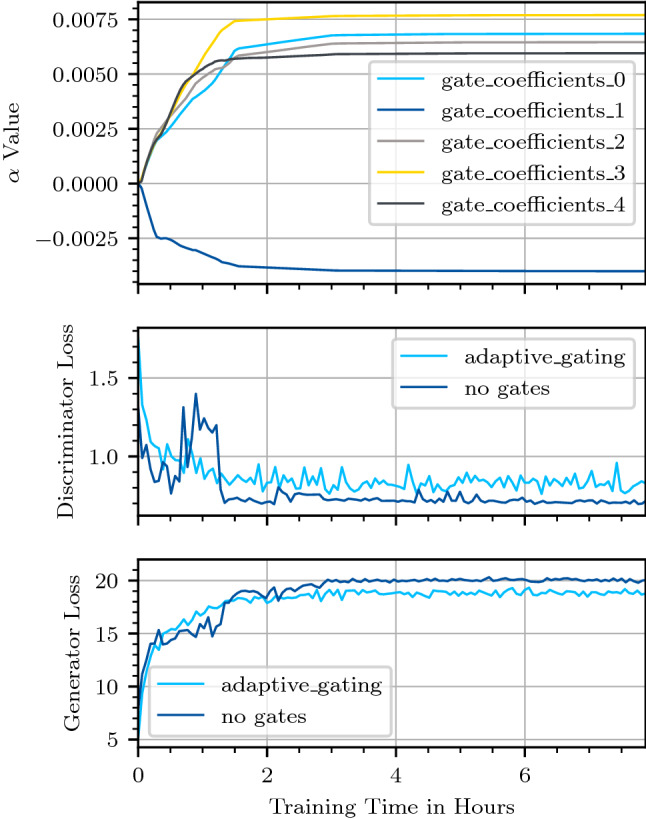
Gate values for the adaptable gates during adversarial training (top) with discriminator (middle) and generator (bottom) training losses. The $\alpha$ values correlate to the adaptive gating (light blue) training shown in the bottom two loss plots, while the dark blue values track the loss for training without any gates included after the residual blocks. It is seen that the gating provides a smoother transition to a stable balance between the generator and discriminator. This subsequently results in better predictions

**Fig. 10:**
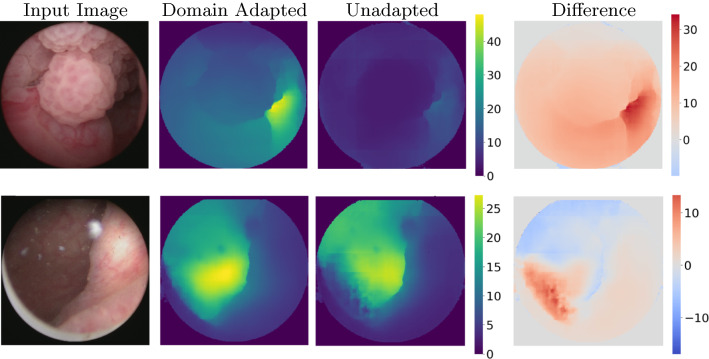
Examples of (perceived) improved depth estimation over synthetic training by using domain adaptation. From left to right: input image, domain adapted depth prediction, unadapted depth prediction using network trained on synthetic data, and difference plot between the two depth predictions. Units are provided in mm and the depth predictions in the second and third columns both use the same scale found in their respective rows

The synthetic data training achieved the lowest validation root mean square error (RMSE) after 22 epochs at 0.878 mm. The weights acquired after this epoch were saved and used for the domain adaptation. To ensure the gating of the domain adaptation functioned as expected, the adversarial training was performed as presented in Sect. 2.4.2 and also repeated with the gating removed. The results of the distribution of the gate coefficients $\alpha _*$ can be seen in Fig. 9. Sample results after training the domain adaptation with gating are shown in Fig. 10. A prediction using the same image before any adaptation is done is provided as well (right depth plot). To get an idea of what is changed between the two depth plots after domain adaptation training, a difference plot between the two depth plots is provided.

**Fig. 11:**
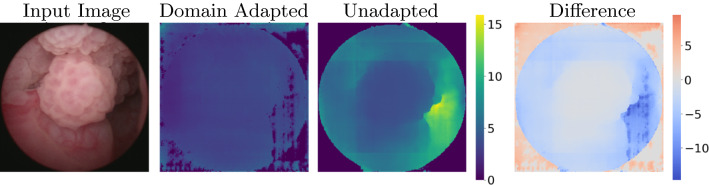
Example of mode collapse during adversarial training for domain adaptation when not using adaptive gating. Units are provided in mm

When comparing to the results in Fig. 10, training the network with the gates removed (ungated training plot from Fig. 9), it is seen that the network almost immediately suffers from a mode collapse and struggles to maintain any information from the provided input image. Instead, only a feasible texture is predicted that can fool the discriminators and the network becomes stuck at this point. The resulting prediction is seen in Fig. 11.

## Discussion

As expected, the convergence of the adversarial training to a stable balance between discriminator training and generator training losses takes longer with the gating but exhibits a much more stable trajectory to the equilibrium in comparison to not using gating. By using an adaptive gating the possibility of needing to restart training due to complete divergence of the network is avoided, which can happen very often when training adversarial networks. In Fig. 10 it is possible to see that including the domain adaptation (left depth plot) produces a more reasonable depth map for the given image. It can be seen that the adversarial training appears to improve upon deciphering the difference between shadows and a darker texture. The checkerboard effect which often appeared when directly applying the synthetic network to real images is effectively eliminated creating a smooth, more continuous depth estimation.

### Limitations

**Fig. 12:**
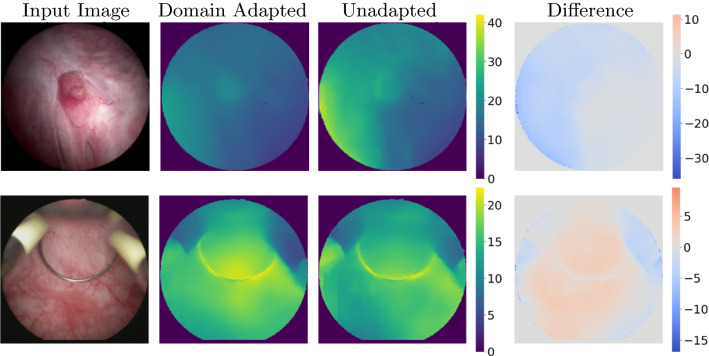
Examples of unimproved depth estimation. Units are provided in mm

It is clear that the proposed domain adaptation through the gated residual blocks accomplishes the intended tasks set forth in this work. Unfortunately, however, as there is no representation for objects such as the resection cutting loop in the current synthetic domain and the simulated polyps do not differentiate much from their surrounding texture, the network really struggles with handling images containing this info. Examples of this are seen in Fig. 12. It is apparent that the network relies purely on the brightness of the tool to determine its distance from the camera and misses the fact that the polyp is not flat since it does not cast a visible shadow in the given image. These problems should be avoidable by including sample images of this data in the synthetic domain such that the network can learn how to map the distinct depth structure of the tool to the latent vector space and that drastically differentiating local texture is an indication of a different structure.

## Conclusion

In this work an improvement on using deep neural networks for monocular depth estimation for cystoscopy was achieved using a two step training approach to limit the problem to a domain transfer between a synthetic and real domain. This was done for the cystoscopic environment inside the human bladder and the work included the construction of a pseudo-realistic bladder environment for the creation of synthetic camera images. Real cystoscopic videos were used for adversarial training to transfer the depth estimation capabilities from the synthetic domain to the real. The training for this was stabilized by restricting the domain adaptation to newly added residual blocks, each with a learnable gating parameter. Results showed an improvement on feasible depth estimations once a domain transfer was done, however, this only worked in scenarios where the synthetic domain was able to provide a similar scene. With these results, it can be concluded that the methods shown enabled depth estimation in a cystoscopic environment and provided a more stable approach to the adversarial training for domain adaptation.

## Data Availability

The source code for this work will be made available at https://github.com/cgtuebingen/cystoscopy_depth.
